# Anything to Get Well

**Published:** 1896-03

**Authors:** Frances J. Dyer


### "Anything to Get Well."

BY FRANCES J. DYER.

How often we hear persons who are partially ill, exclaim, in tones as if they felt themselves abused: "I would do anything to get well." Yet, when we come to probe their mode of living, we find that self-gratification in some form, and usually that of the appetite, lies at the root of their ailments. The sufferer seeks change of scene and climate, flees to Nice or Los Angeles, or wherever the fountain of health is supposed to be situated, ignoring the fact that the fundamental change must begin with themselves and not with external conditions.

Perhaps the system cannot receive coffee without detriment. Yet, let the physician prohibit its use and at once the patient cries out: "O doctor, don't ask me to give up my coffee. Why, I couldn't make a meal without that!"

Or perhaps an excess of sweets is undermining the constitution. We know a woman who buries her morning cereal with sugar, finishes her breakfast with doughnuts or cakes, uses three times as much sweetening in her beverages as she ought, and as a consequence is troubled with nervousness, constipation, irritability and sleeplessness. Friends remonstrate in vain. She resents interference and insists that her diet has no connection whatever with her condition. She will take medicine when prescribed by a physician, but she will not deny herself the pleasure of eating sweets.

A certain business man in Boston is what is called "a high liver." He uses neither wine nor tobacco in any form, but his table is loaded with a variety of the choicest food. He claims that his active life demands a generous diet and that so long as the viands are properly cooked no harm can result from what—to speak plainly—is refined gluttony. But every few months he has an acute attack of intestinal disorder accompanied by excruciating suffering.

The wise old specialist who is called to attend him, and who charges an enormous fee for his services, prescribes but a modicum of medicine, and limits his patient to a strict diet of dry toast and water for several days. Nature thus has a chance to throw off the superfluity which has deranged the system.

A teacher in the sciences in a private school in New York was demonstrating to her pupils the indigestibility of a certain toothsome dish, when one of the young ladies said deprecatingly: "Oh, but it tastes so good. You couldn't ask us to give up eating that."

Such cases could be multiplied indefinitely, but these are sufficient to show that people are willing to do "anything to get well"--or to keep well--except to surrender their pet tastes in food and drink. If they do not break down altogether in health they are only half well, and are forever making some outward application or taking some internal remedy to improve their condition.

The price of health is obedience to natural laws and that often means the sacrifice of desires which are in danger of enslaving the life with fetters like iron. But law will not compromise. It says: "Eat and drink indiscreetly if you will, give the rein to passion, cheat your lungs out of their quota of fresh air, dress unhygienically; but know that for all these things, sooner or later, you will be brought into judgment."

With the greater intelligence which prevails to-day in respect to dietetics there is need also of developing more power of self-control over the appetite. Lack of this is like the little crevice in the dyke which lets in a devastating flood of physical ills.—*American Kitchen Magazine.*

---

PROF. GRAY has recently adopted a very satisfactory method of using gutta-percha. After drying the cavity he saturates it with common resin cut in chloroform and then presses in heated gutta-percha. It adheres to the wall like cement and does not pull away. He has found it very satisfactory in the mouths of his own children where he has the opportunity of observing it closely.

